# A Real-Time Capable Software-Defined Receiver Using GPU for Adaptive Anti-Jam GPS Sensors

**DOI:** 10.3390/s110908966

**Published:** 2011-09-19

**Authors:** Jiwon Seo, Yu-Hsuan Chen, David S. De Lorenzo, Sherman Lo, Per Enge, Dennis Akos, Jiyun Lee

**Affiliations:** 1 Department of Aeronautics and Astronautics, Stanford University, 496 Lomita Mall, Stanford, CA 94305, USA; E-Mails: jwseo@cs.stanford.edu (J.S.); dsd@stanford.edu (D.S.D.); daedalus@stanford.edu (S.L.); penge@stanford.edu (P.E.);; 2 Department of Electrical Engineering, National Cheng Kung University, 1 University Road, Tainan 70101, Taiwan; E-Mail: shinge@stanford.edu; 3 Department of Aerospace Engineering Sciences, University of Colorado, 1111 Engineering Drive, Boulder, CO 80309, USA; E-Mail: dma@colorado.edu; 4 Department of Aerospace Engineering, Korea Advanced Institute of Science and Technology, 335 Gwahangno, Yuseong-gu, Daejeon 305-701, Korea

**Keywords:** Global Positioning System (GPS) sensor, software-defined radio, controlled reception pattern antenna (CRPA), radio frequency interference, parallel processing, Graphics Processing Unit (GPU)

## Abstract

Due to their weak received signal power, Global Positioning System (GPS) signals are vulnerable to radio frequency interference. Adaptive beam and null steering of the gain pattern of a GPS antenna array can significantly increase the resistance of GPS sensors to signal interference and jamming. Since adaptive array processing requires intensive computational power, beamsteering GPS receivers were usually implemented using hardware such as field-programmable gate arrays (FPGAs). However, a software implementation using general-purpose processors is much more desirable because of its flexibility and cost effectiveness. This paper presents a GPS software-defined radio (SDR) with adaptive beamsteering capability for anti-jam applications. The GPS SDR design is based on an optimized desktop parallel processing architecture using a quad-core Central Processing Unit (CPU) coupled with a new generation Graphics Processing Unit (GPU) having massively parallel processors. This GPS SDR demonstrates sufficient computational capability to support a four-element antenna array and future GPS L5 signal processing in real time. After providing the details of our design and optimization schemes for future GPU-based GPS SDR developments, the jamming resistance of our GPS SDR under synthetic wideband jamming is presented. Since the GPS SDR uses commercial-off-the-shelf hardware and processors, it can be easily adopted in civil GPS applications requiring anti-jam capabilities.

## 1. Introduction

Since Global Navigation Satellite System (GNSS) sensors (e.g., Global Positioning System or GPS of the US [1,2], Galileo of Europe, GLONASS of Russia, and Compass of China) are widely used in many fields, including navigation, surveillance and precise timing, their vulnerability to radio frequency interference (RFI) is drawing significant attention. GPS RFI [3–5] is especially problematic for GPS-based safety-of-life services such as aviation. For example, GPS RFI was detected during the installation of a Ground-Based Augmentation System (GBAS) [6–10] ground monitor at Newark airport (EWR) on 23 November 2009 [11]. The installed GBAS can support GPS-based precision landing approach of aircraft down to 200 ft above the runway. Since then, many other GPS RFI events have been detected at Newark airport and the certification of that GBAS has been delayed. The RFI sources turned out to be personal “privacy jammers” in vehicles on the nearby highway, which are designed to overwhelm weak GPS signals by transmitting signals in the GPS L1 frequency (1.575 GHz).

In order to guarantee the integrity and availability of civil aviation under GPS RFI, the United States Federal Aviation Administration (FAA) has recently initiated an effort called Alternative Position Navigation and Timing (APNT) to provide a navigation system for aviation that can provide many of the operational capabilities that will be enabled by GPS [12,13]. The aim of APNT is to provide robustness for the US National Airspace to GPS outages due to RFI and other events. Among various GPS anti-jamming technologies, an adaptive beamsteering GPS receiver has been proposed for robust time synchronization between terrestrial assets used by APNT, such as Ground Based Transceivers (GBTs), even under GPS RFI [14,15].

**Figure 1.**
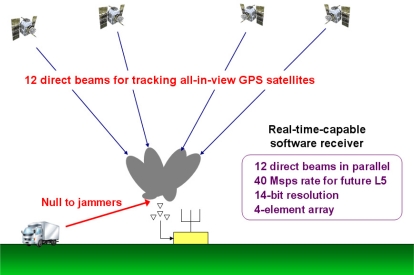
Illustration of the functionality of our GPS SDR for processing a four-elememt controlled reception pattern antenna (CRPA) array. The receiver adaptively rejects jammers while steering high gains toward all GPS satellites in view.

Since ground stations are stationary, in principle one tracked satellite is adequate for time transfer. However, if a beamsteering GPS receiver can steer directed beams towards all GPS satellites in view and reject RFI adaptively as presented in this paper (Figure 1), it could be potentially used for many other applications such as hardening GBAS ground facilities. Mitigation of RFI for generic GPS applications using adaptive antenna arrays has been previously studied (e.g., [16–18]). In order to use a controlled reception pattern antenna (CRPA) array for high-integrity GPS applications such as aircraft landing guidance [19], the system should satisfy four quality requirements which are accuracy, integrity, availability, and continuity [20]. Several researchers have studied the feasibility of using CRPA for high-integrity GPS applications [21–23].

The development of GPS software-defined radio (SDR) [24,25] has been beneficial for studying adaptive beamsteering GPS receiver technology as well [26]. A software implementation of a GPS receiver is more desirable than a traditional hardware implementation using field-programmable gate arrays (FPGAs) or application-specific integrated circuits (ASICs) for multiple reasons. First, such an implementation has higher flexibility and shorter development time, allowing new algorithms to be quickly implemented and tested. Second, a software implementation that can run on commercial off-the-shelf (COTS) general-purpose processors makes the technology more cost effective and accessible for the civil consumer market. Although a real-time GPS SDR requires extensive computational power, real-time GPS L1 SDRs using COTS general-purpose processors has been developed by several researchers (e.g., four tracking channels for GPS in 2001 [27] and 24 tracking channels for GPS and Galileo in 2006 [28]).

However, development of a real-time GPS SDR for CRPA is still challenging because adaptive GPS beamsteering processing requires much more computational power than a conventional GPS receiver. We are not aware of a previous real-time GPS SDR development for CRPA with all-in-view satellite tracking capability. Chen *et al.* [29] presented a real-time beamsteering GPS SDR for robust time transfer for APNT. Since the receiver is only for a time transfer application, it makes a direct beam toward a single satellite (*i.e.*, single-beamsteering-channel SDR). The receiver processes intermediate frequency (IF)-sampled data with 16 Msps (mega samples per second) rate (16 Msps real samples). This sampling rate is sufficient for GPS L1 C/A signals because L1 C/A-code bandwidth is about 2 MHz, but it is not enough for future GPS L5 signals whose code bandwidth is about 20 MHz. Further, 2-bit sampling resolution of this previous work does not provide necessary dynamic range for anti-jamming applications. In this paper, we present a novel SDR architecture for GPS anti-jamming reference station receivers with CRPA using COTS general-purpose parallel processors. The GPS SDR for CRPA presented in this paper has capabilities far exceeding those described in this previous work.

We achieved required computational power by parallel processing on a new generation general-purpose Graphical Processing Unit (GPU) and a novel design scheme (see [30] for the evolution of GPUs for general-purpose computing). Among many applications, GPUs can be used for sensor systems which require significant processing power (e.g., [31]) because GPUs provide very high computational throughput from their massive number of threads. GPU-based GPS SDRs without beamsteering capability have been previously developed by several researchers [32–35]. Knezevic *et al.* [32] developed an 8-channel GPS SDR capable of processing 40 Msps and 8-bit resolution data in real time using a single-core 3.0 GHz CPU and an NVIDIA GeForce 8800 GTX GPU. Hobiger *et al.* [33] developed a real-time GPS SDR supporting 12 channels with 8 Msps and 4-bit resolution data using an Intel Core 2 Q9450 CPU and an NVIDIA GeForce GTX 280 GPU. Cailun *et al.* [34] stated that they developed a GPS SDR running 150 channels with 5 Msps and 14-bit resolution data using an Intel Xeon 5150 CPU and an NVIDIA GeForce GTX 285 GPU, however their paper does not present the design details. All these developments in [32–34] utilized GPUs for correlation operations for code tracking. On the other hand, Pany *et al.* [35] used an NVIDIA GTX 480 GPU to expedite GNSS signal acquisition. The GPUs that have been used by these previous studies are summarized in Table 1.

Our GPS SDR for CRPA presented in this paper has 12 beamsteering channels for all-in-view satellite tracking (Figure 1), and it processes IF-sampled data at a 40 Msps rate (20 Msps inphase and quadrature samples) which enables future GPS L5 signal tracking whose bandwidth is ten times wider than the L1 C/A-code bandwidth. Further, our receiver processes digitized samples with 14-bit resolution. Although most commercial GPS receivers process 2-bit resolution data, more dynamic range is required for anti-jamming applications. As a very rough comparison, the GPU-based SDR in [34] effectively processes 750 Msps (5 Msps data and 150 tracking channels), but our GPU-based SDR for CRPA effectively processes 2,400 Msps (40 Msps data and 60 tracking channels because 12 channels for each antenna element of a four-element array and additional 12 channels for beamsteering). In addition, our receiver calculates signal covariance matrix for adaptive interference rejection which requires additional computational power. To the authors’ knowledge, a real-time capable GPS SDR for CRPA with comparable functionalities on general-purpose CPU and GPU has not been previously demonstrated.

This paper is organized as follows: Section 2 presents our GPS SDR architecture and discusses the computational challenges in detail. Our SDR design with a real-time phase calibration feature considers a GPS reference station receiver under RFI. Since a CRPA array is more voluminous than a conventional patch antenna, GPS infrastructure receivers such as differential GPS (DGPS) reference station receivers are more likely to support CRPA than mobile users. Section 3 explains our implementation details using an Intel Core i7 950 CPU and an NVIDIA GTX 480 GPU. Although a modern GPU can easily run thousands of threads in parallel, each GPU thread is not as powerful as a CPU thread. Roughly speaking, a CPU is more powerful for serial processing and a GPU is more powerful for parallel processing. In our SDR design, computational loads are balanced between CPU and GPU by considering their strengths. After discussing anti-jamming performance of the GPS SDR for a four-element CRPA in Section 4, conclusions are given in Section 5.

## 2. GPS SDR Architecture for CRPA Processing and Computational Challenges

**Figure 2.**
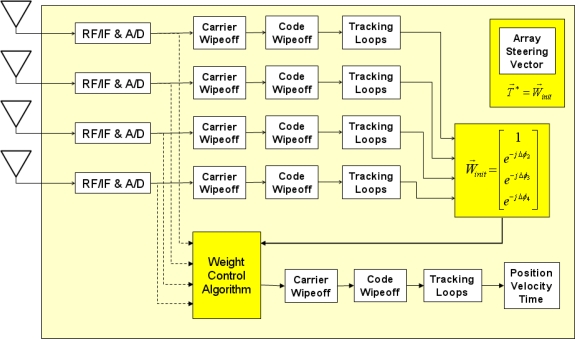
Inter-channel phase alignments using independent satellite tracking channels for each antenna element. Different array steering vectors are obtained at each epoch for different satellites (*i.e.*, 12 different steering vectors for our GPS SDR supporting 12 beamsteering channels).

Adaptive array processing starts from phase calibration between antenna elements for each satellite tracking channel. Once the carrier phases of all antenna elements are aligned, signal-to-noise-density-ratio (C/N_0_) of the corresponding satellite channel can be enhanced by combining all phase-aligned signals (*i.e.*, high antenna gain, or beam, would be formed toward the satellite). One method of phase alignment is to use array synthesis techniques that consider array orientation, baseline geometry, satellite constellation, line biases, filter delays, etc. Furthermore, antenna anisotropy and mutual electronic coupling between antenna elements should be considered for precise phase alignment. In our experience this method is considerably more difficult than our method illustrated in Figure 2.

Figure 2 shows phase alignment of one satellite channel as an example. Four antenna elements independently track the same satellite. Then, a carrier phase of each antenna for the satellite (*i.e.*, *ϕ*_1_, *ϕ*_2_, *ϕ*_3_, and *ϕ*_4_) is obtained from its tracking loop. Now phase alignment can be done by simple phase rotation with phase difference between each antenna and a reference antenna (*i.e.*, Δ*ϕ*_1_ = *ϕ*_1_ – *ϕ*_1_ = 0, Δ*ϕ*_2_ = *ϕ*_2_ – *ϕ*_1_, Δ*ϕ*_3_ = *ϕ*_3_ – *ϕ*_1_, and Δ*ϕ*_4_ = *ϕ*_4_ – *ϕ*_1_). This phase alignment method is conceptually straightforward, but it has several limitations. For each satellite tracking, five independent tracking channels are required for a four-element array system (*i.e.*, four tracking channels for four antenna elements and one tracking channel for a synthesized beamsteering signal). Thus, five-times more computational power than for a conventional receiver is required. This method is applicable for mobile GPS receivers with CRPA to reduce multipath effects by forming high antenna gains in the directions of direct signal paths from satellites. However, this method may not be useful for mobile users under strong RFI because tracking channels for a single antenna element can lose lock under RFI, and thus phase differences between antenna elements cannot be calculated on-the-fly.

As mentioned in Section 1, the applications of our anti-jamming GPS SDR for CRPA are GPS ground station receivers such as time transfer receivers for GBTs, GBAS ground facility receivers, and DGPS reference station receivers. Since these receivers are stationary, the simple phase alignment method is still applicable even under RFI. When RFI does not exist, which is a usual condition, an array steering vector, T*, for each satellite can be obtained by independently tracking each antenna element as in Figure 2. Then, the array steering vectors are stored as a lookup table with time stamps. Since GPS satellite ground tracks repeat in a known basis, the stored array steering vectors can be used for a stationary GPS receiver when RFI makes the on-the-fly phase alignment impossible. Again, this method is not adequate if a GPS receiver with CRPA is not stationary.

**Figure 3.**
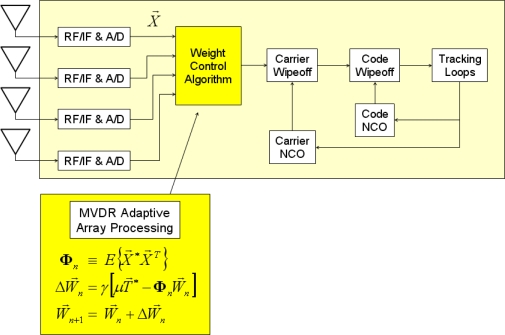
Adaptive RFI cancellation by MVDR processing with given array steering vectors from the phase alignment in Figure 2.

Once array steering vectors are given, an adaptive RFI cancellation algorithm is applied as in Figure 3. Among two algorithms reviewed in [37], the minimum-variance distortionless-response (MVDR) algorithm [38] is applied in this paper. The optimal weight vector, **W**, in MVDR processing is estimated by Equation (1). The measurement vector, **X**, is composed of the digitized signals right after the analog-to-digital converter in our implementation, but it can be the sample vector after carrier wipeoff for pre-correlation adaptation or the inphase and quadrature prompt correlator output vector for post-correlation adaptation [Φ in Equation (1) is a signal covariance matrix and *μ* is a signal power scaling factor. The ^*^ operator represents complex conjugate]:

(1) $$\begin{array}{l} \text{Φ}=E\left[{\text{X}}^{*}{\text{X}}^{T}\right]\\ \text{W}=\mu {\text{Φ}}^{-1}{\text{T}}^{*} \end{array}$$

For computational efficiency, a propagating approach to update weight vector by Equation (2) is implemented in our SDR [37]. This approach does not require to calculate an inverse of Φ (*γ* is a misadjustment parameter which controls convergence speed and steady-state misadjustment):

(2) $$\begin{array}{l} {\text{W}}_{n+1}={\text{W}}_{n}+{\Delta \text{W}}_{n}\\ {\Delta \text{W}}_{n}=\gamma \left[\mu {\text{T}}^{*}-{\text{Φ}}_{n}{\text{W}}_{n}\right] \end{array}$$

Before discussing the required computational power for our GPS SDR for a four-element CRPA, the number of multiplications and additions for a conventional GPS SDR with a single antenna is calculated as follows (see Table 2 for summary). The carrier wipeoff is a complex multiplication of digitized input signals and receiver-generated carrier samples (this is not simply two real multiplications because input signal is a complex signal with inphase and quadrature samples). Since one input sample takes four multiplications and two additions of real numbers for the carrier wipeoff as shown in Equation (3), the total number of multiplications and additions for *N* inphase samples and *N* quadrature samples per millisecond is 6*N*:

(3) (a+jb)(c+jd)=(ac−bd)+j(ad+bc)

The code wipeoff is the correlation of signals after the carrier wipeoff and receiver-generated C/A code replica samples. A correlation of *N* input inphase samples and *N* code replica samples takes *N* multiplications and *N* − 1 additions. Since *N* is much larger than 1, approximately 2*N* multiplications and additions are required for inphase samples. Usually, a GPS receiver correlates incoming signals with an early, a prompt, and a late code replica. Thus, 6*N* operations are performed for inphase samples. The same operations are performed for quadrature samples, so the total number of operations is 12*N*. Considering both carrier and code wipeoff, 6*N* + 12*N* = 18*N* multiplications and additions are required every millisecond for a single tracking channel of a conventional GPS SDR, where *N* is the number of inphase or quadrature samples per millisecond. For a 12-channel SDR, 12 × 18*N* = 216*N* multiplications and additions are required. Other operations such as tracking and position calculation are neglected in this simple analysis because those operations are not as computationally expensive as the carrier and code wipeoff. The carrier and code generation operations are not included in the number of operations calculation because lookup tables are used instead of generating code and carrier on-the-fly.

In our GPS SDR for a four-element CRPA, five tracking channels are necessary for each beamsteering channel due to the on-the-fly phase calibration. Thus, the cost of carrier and code wipeoff is 5 × 216*N* = 1,080*N*. For beamforming, input data from four antennas should be combined together with proper weights. Weighting is complex multiplications as in Equation (3), and *N* inphase and *N* quadrature samples per antenna requires 6*N* multiplications and additions for weighting. Thus, the weighting of four-antenna data costs 4 × 6*N* = 24*N*. After weighting each antenna data, all weighted data need to be added together, which requires 3*N* additions for inphase samples and 3*N* additions for quadrature samples per beamsteering channel. Thus, 12 × (24*N* + 3*N* + 3*N)* = 360*N*. operations are required for 12 beamsteering channels having 12 different weight vectors.

The MVDR processing requires signal covariance matrix calculation. The measurement vector **X** in Figure 3 and Equation (4) is a 4-by- *N* complex matrix. The calculation of AB*^T^* in Equation (4) takes approximately 32*N* multiplications and additions (N multiplications and N-1 additions for each element of AB*^T^* which has 16 elements), but the calculation of AA*^T^* or BB*^T^* takes about a half operations because of its symmetry. Thus, X^*^X*^T^* costs approximately 16*N* + 16*N* + 32*N)* = 64*N*.

(4) $${\text{X}}^{*}{\text{X}}^{T}=(\text{A}-j\text{B})({\text{A}}^{T}+j{\text{B}}^{T})=({\text{AA}}^{T}+{\text{BB}}^{T})+j({\text{AB}}^{T}-{\text{A}}^{T}\text{B})$$

Therefore, the total number of multiplications and addition for the carrier and code wipeoff, data synthesis, and covariance calculation for our GPS SDR for a four-element CRPA is approximately 1,080*N* + 360*N* + 64*N* = 1,504*N*. This required number of operations per millisecond is significantly larger than the number of operations of a conventional 12-channel and single-antenna GPS SDR, which is 216*N* (Table 2). Since GPS L5-code bandwidth is ten times wider than GPS L1 C/A-code bandwidth, the sampling rate for L5 processing should be ten-times higher and thus the number of digitized samples per millisecond would be ten-times more. Our GPS SDR is designed to process 40 Msps for future L5 processing. Hence, the required computational cost of our SDR for CRPA is about 70-times higher than a 12-channel single-antenna GPS SDR processing 4 Msps L1 C/A signals as shown in Equation (5):

(5) $$\frac{1504N_{L5}}{216N_{L1}}=\frac{1504\times 10N_{L1}}{216N_{L1}}=69.63$$

If a GPS SDR processes only 2-bit resolution data, the bitwise-parallel correlation algorithm in [39] can reduce the computational burden. The bit-wise parallel correlation is known to be about twice as fast as a conventional integer correlation. However, an anti-jam GPS SDR for CRPA requires more dynamic range and our SDR processes 14-bit resolution data. In this case, the bitwise-parallel correlation scheme does not provide any benefit.

Although current CPUs in the market are very powerful, it is very unlikely we will have a CPU whose single thread can run 70 GPS L1 SDRs simultaneously in the foreseeable future. Even modern multi-threaded C/C++ codes on a quad-core CPU in [29] could not achieve this much computational power. As a more powerful general-purpose processor specifically designed for parallel processing, a GPU can share significant computational burden as a co-processor. Nevertheless, GPU-based GPS SDRs in the literature (Table 1) do not seem to demonstrate enough computational power for the CRPA processing explained in this section. Although a rigorous comparison between different GPS SDR performances is not possible without profiling the source codes, rough comparisons can still be made. For example, the 8-channel, 40 Msps GPS SDR in [32] is not enough for the CRPA processing of this paper which requires correlation operations of 60 channels with 40 Msps data. The 150-channel, 5 Msps GPS SDR in [34] can simultaneously run roughly less than 20 GPS L1 SDRs with 12 channels each, but it is not enough for the CRPA processing which requires the computational cost of about 70 GPS L1 SDRs [Equation (5)].

However, these GPUs have compute capability 1.x (Table 1) and these SDR performances could be enhanced by using new generation GPUs of compute capability 2.x. Although backward compatibility is guaranteed, performance enhancement by a new generation GPU is limited if unmodified source codes designed for a previous generation GPU is used, because the new generation GPUs of compute capability 2.x have a very different architecture called Fermi architecture [40]. In order to take full advantage of Fermi architecture, we specifically designed our GPU-based GPS SDR for CRPA in a way to maximize the utility of new resources from the new GPU architecture. The GPU of compute capability 2.0 used in this work is NVIDIA GeForce GTX 480 which was released in March 2010.

Although a GPU can provide more computational throughput than a CPU, certain tasks may not be readily parallelizable for GPU processing. More important, it is not desirable to idle a CPU while a GPU performs its tasks. Thus, a wise use of CPU in parallel with GPU can always improve the SDR performance. In our design, the computational burden is shared by multi-threading on a quad-core CPU. In this way, a multi-core CPU provides thread-level parallelism in addition to the hardware parallelism as a different hardware from GPU. The novel GPS SDR implementation by parallel processing on CPU and GPU is detailed in the next section.

## 3. GPS SDR Design for CRPA Using General-Purpose Parallel Processors

### 3.1. Hardware Setup

**Figure 4.**
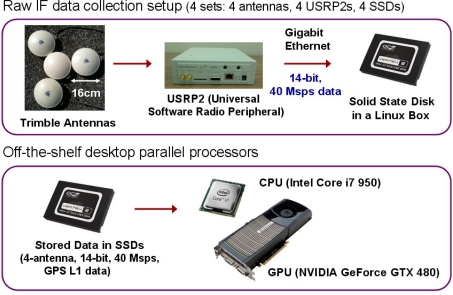
Hardware setup for GPS SDR for four-element CRPA processing.

The entire receiver processing after the analog-to-digital converters in Figures 2 and 3 is performed on general-purpose processors in our SDR implementation. Four Trimble Zephyr antennas are used as an *ad hoc* CRPA array (Figure 4). GPS L1 signals from four antennas are down-converted to baseband and digitized by four commercial digitizers (Universal Software Radio Peripheral 2, or USRP2, from Ettus Research LLC).

For raw data recording, the sampling rate of each USRP2 is set to 40 Msps (20 Msps inphase and quadrature samples). Each sample size is 2 byte with 14-bit resolution. Although a 40 Msps rate is adequate for GPS L5 signal processing, only one GPS satellite is transmitting a healthy L5 signal as of July 2011. Thus, it is not possible to demonstrate 12-channel GPS L5 SDR processing with live signals. As an alternative, we process L1 signals with 40 Msps rate to demonstrate the SDR’s computational capability for future real-time L5 processing. Otherwise, it is unnecessary to process L1 signal with 40 Msps rate because L1 C/A-code bandwidth is only 2 MHz. However, it is slightly optimistic to directly transfer the 40 Msps L1 C/A-code results in this paper to L5 because L5 has data and pilot signals, longer codes, and a secondary code, which are not considered in the L1 C/A-code tracking.

The digitized samples are streamed via gigabit Ethernet to solid state disks (SSDs) within computers. SSDs are used because streamed data rate from each antenna is 80 MB/s which is higher than the writing rate of conventional hard disks. After storing four-antenna data in SSDs, the real-time computational capability of our GPS SDR for CRPA is demonstrated by replaying the stored data faster than real time. The COTS general-purpose parallel processors (CPU and GPU) used for the GPS SDR are shown in Figure 4.

### 3.2. GPU-Based Parallel Correlator

**Figure 5.**
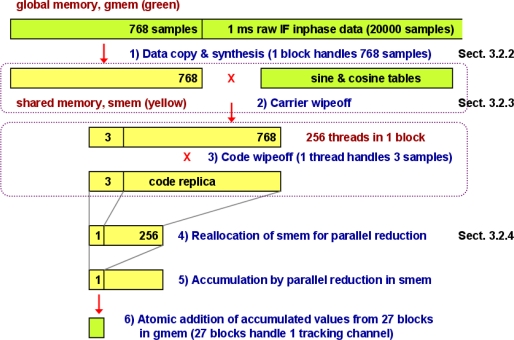
GPU-based parallel correlator design. The operations of single thread block (256 threads) on inphase data with a prompt C/A code replica are illustrated here. Thread blocks perform similar operations every millisecond on quadrature data with an early and a late code replica as well. Total 27 thread blocks are needed for each tracking channel, and total 60 tracking channels are needed for the CRPA processing.

The basic design idea of the GPS SDR for CRPA is to maximize parallelism in receiver architecture. As discussed in Section 2, the carrier and code wipeoff has the biggest computational load (1,080*N* multiplications and additions every millisecond, in other words 1,080*N*/1,504*N* ≈ 72% of total computational load, *N* = 20,000 in our case) but it can be highly parallelized. Compute Unified Device Architecture (CUDA) is NVIDIA’s parallel computing architecture, and we use a CUDA-enabled GPU to perform the carrier and code wipeoff operations. Figure 5 illustrates our GPU-based parallel correlator. Our GPU code is written in CUDA C programming language [36,41].

The GTX 480 GPU used in this work has 15 multiprocessors and 480 CUDA cores (Table 1). Since each multiprocessor of GPUs of compute capability 2.x can have 1,536 active threads (Table 3), the GTX 480 GPU can have 1,536 × 15 = 23,040 active threads.

However, this maximum possible GPU occupancy may not be achieved for various reasons. The GPU occupancy is determined by the number of threads per thread block, the number of registers used per thread, and the allocated shared memory per thread block. Once these three design parameters are determined, the GPU occupancy of the design can be calculated using the CUDA GPU occupancy calculator [42]. After several design iterations, we assign 256 threads (or eight warps) per thread block and five thread blocks per multiprocessor. In CUDA, threads are executed in groups of 32 threads. This 32-thread group is called a “warp”. A multiprocessor of GTX 480 can have 48 active warps (Table 3). Thus, when each thread block has eight warps and six thread blocks are assigned to each multiprocessor, a 100% GPU occupancy (*i.e.*, all 48 warps in use) can be achieved. However, our design is limited by the number of registers per multiprocessor (the physical limit is 32 K registers per multiprocessor as shown in Table 3) and only five thread blocks can be assigned to each multiprocessor. Hence, 5 block / multiprocessor×8 warp / block = 40 warp / multiprocessor are assigned. The GPU occupancy in this case is 40 warp / 48 warp = 83.33%.

If we can reduce the number of assigned registers per thread, the occupancy can be increased, but it is generally difficult to reduce the number of register variables in source code while performing the same task. The maximum number of registers per thread can be manually forced using ‘–maxrregcount’ flag at compile time. Although this flag may force to decrease register usage and increase the occupancy, it does not always improve performance for two reasons. First, spilled registers are stored in much slower local memory. Second, higher occupancy above a certain point does not improve performance (Chapter 4 of [43]). We force 24 registers per thread using this flag and it results in the best performance in our case.

According to Section 3.2.6 of [43], as many as 24 warps (768 threads) per multiprocessor (*i.e.*, 50% occupancy) are required to completely hide the latency of registers’ read-after-write dependencies. If occupancy is more than 50%, it may not be necessary to further optimize parameters to obtain even higher occupancy. Note that the GTX 480 GPU has 480 CUDA cores (Table 1), which is theoretically capable of performing 480 16-bit floating-point operations (multiply, add, or multiply-and-add) per clock cycle. With the 83.33% occupancy, one multiprocessor has 1,536 × 83.33% = 1,280 active threads, and the GTX 480 GPU with 15 multiprocessors (Table 1) has 1,280 × 15 = 19,200 active threads. Thus, it is clear that CUDA cores cannot perform operations on all active threads simultaneously at the same clock cycle. However, it is important to generate large number of active threads to hide memory latency. In this way, CUDA cores would continuously have enough threads to process while other threads wait for memory reading. (Hiding memory latency in GPU will be further discussed in Section 3.4.)

#### 3.2.1. Memory Space on a CUDA Device

**Figure 6.**
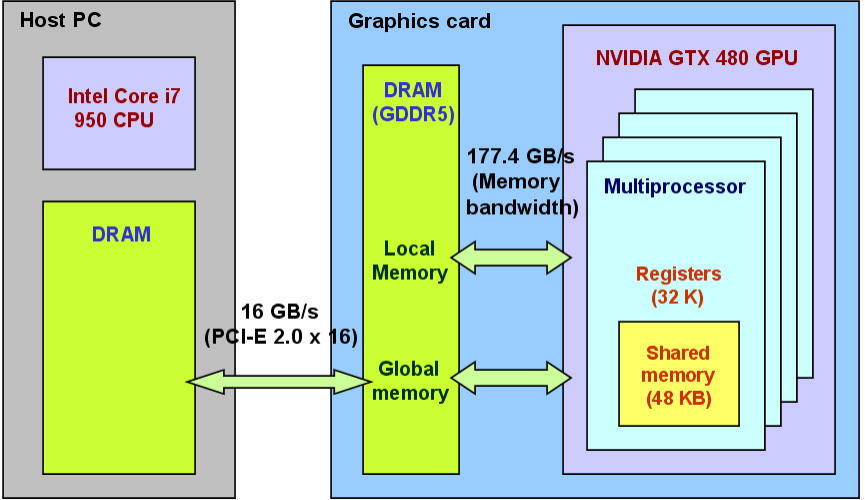
Memory space on a host computer and a CUDA device. Constant memory and texture memory on a CUDA device are not shown here because they are not used in our SDR implementation.

Before explaining the correlator design in Figure 5, we briefly review the memory space on a CUDA device. Figure 6 illustrates a host computer and a graphics card, or “device”, with the GTX 480 GPU. In order to process data in the memory of a host computer using GPU, the data must be copied over to the global memory of a device. The data bus for the GTX 480 GPU is PCI-E 2.0 × 16 whose maximum bandwidth is 16 GB/s. Once the data is copied from host memory to device global memory, multiprocessors in GPU access the data in global memory for processing. The theoretical maximum global memory bandwidth of the GTX 480 GPU is 177.4 GB/s [44]. This number is calculated in Equation (6) based on its specification. Its memory interface width is 384 bit. Memory clock is 1,848 MHz and it is a double data rate (DDR) memory:

(6) $$384\;\text{bit}\times \frac{1}{8}\text{Byte}/\text{bit}\times 1848\;\text{MHz}\times 2=177.4\;\text{GB}/s$$

Local memory is also placed in DRAM and it is for large structures and arrays or spilled registers. If a GPU code, or “kernel”, requests more than available registers, those variables are placed in local memory.

DRAM access has high latency due to limited bandwidth, but on-chip memory access is much faster. The latency of uncached on-chip shared memory is roughly 100 times lower than the latency of global memory (Section 3.2.2 of [43]). On-chip registers also have low latency. Therefore, a basic optimization strategy is to minimize high latency memory access such as host-to-device memory copy and global memory access. Memory latency is not usually a problem for CPU because it has a sophisticated and optimized cache hierarchy. GPU is optimized for high processing throughput instead and it has weakness on tasks requiring high memory access but low computational intensity. In terms of processing throughput, the theoretical maximum throughput of 32-bit floating-point operations on the GTX 480 GPU is 1.34 Tflops (Tera floating-point operations per second), which is about ten times higher than the throughput of modern CPUs (See Figure 1-1 of [36]). The theoretical maximum throughput of GTX 480 is calculated in Equation (7) based on its specifications [44]. Each CUDA core can perform one 16-bit multiply-and-add operation (*i.e.*, two 16-bit floating-point operations) per clock cycle:

(7) 480 cores×1401 MHz×2 flop/core/cycle=1.34 Tflops

#### 3.2.2. Data Copy and Synthesis for Beamforming

Every millisecond, raw inphase and quadrature samples in the host memory are copied to the device global memory for the carrier and code wipeoff by GPU (Figure 5). The latency of this host-to-device memory copy can be hidden by running the GPU direct memory access (DMA) engine and compute engine in parallel, which will be discussed in Section 3.3. However, the global memory access latency is still a major design concern. General optimization practice to minimize global memory access is to access global memory once and store the read data in on-chip shared memory for later processing because shared memory access is much faster. As shown in Table 3, a new generation GPU of 2.x compute capability has three times larger shared memory per multiprocessor than previous generations, but it is still a very limited resource (48 KB per multiprocessor). In our implementation, five thread blocks are assigned in each multiprocessor, so each thread block can use up to 9.6 KB shared memory. Thus, we copy 768 inphase samples and 768 quadrature samples, and 788 C/A code values (total 9.3 KB) from global memory to shared memory every millisecond (Figure 5). Note that 20 more samples, which is equivalent to one C/A code chip, are copied for C/A code than the number of inphase or quadrature samples because we need an early and a late C/A code replica, which are separated by one C/A code chip, in addition to a prompt replica.

It is also an important optimization practice to minimize divergent warps (Section 6.1 of [43]). If threads within the same warp follow different execution paths, processing throughput would be impacted. For this reason, the number of assigned samples (768 samples) is taken as a multiple of the number of threads (256 threads) in a thread block so that every thread can perform identical operations on different data samples assigned to each thread. Although 788 C/A code values are stored in shared memory, appropriate 768 samples are selected as an early, a prompt, and a late code replica. If we copy 256 more samples of inphase, quadrature, and C/A code to shared memory, it does not fit into 9.6 KB limit.

During the data copy from global memory to shared memory, aligned coalesced global memory access is highly desirable (Section 3.2.1 of [43]). Coalesced memory access is achieved by thread indexing so that adjacent threads can access adjacent 16-bit integer samples in global memory (the inphase and quadrature samples in global memory are 16-bit integers from analog-to-digital converters. These 16-bit integers are copied to shared memory as 32-bit floating-point numbers for further GPU processing). However, aligned access with a 128-byte memory segment is not guaranteed in our design. Since each tracking channel experiences different Doppler frequency, the C/A code boundary of each channel in the inphase and quadrature sample stream is not aligned. Thus, we need to copy 1 ms samples (20,000 inphase and 20,000 quadrature samples) for each tracking channel with different starting point in global memory and the starting point may not be aligned with a 128-byte memory segment. If the global memory access is not aligned with a 128-byte segment, two 128-byte memory transactions for each 128-byte access by a warp would be performed by the default setting of compute capability 2.x devices (Section F.4.2 of [36]).

The synthesis of raw data from four antenna elements with proper weights is an essential part of the GPS CRPA processing (Figures 2 and 3). Following the discussion in Section 2, the data synthesis would take 360*N* /1504 *N* ≅ 24% of total computational load if the whole processing is serialized (Table 2). In our implementation, the data synthesis is done in parallel by massive number of active threads (19,200 threads as shown earlier in Section 3.2). This parallelism is realized by multiplying appropriate weights to the inphase and quadrature samples by each thread while the data is copied from global memory to shared memory. Since the memory copy operation is already performed in parallel by massive number of threads, the data synthesis can be also performed in parallel by adding simple multiplications and additions in each thread during the copy operation.

#### 3.2.3. Carrier and Code Wipeoff

After the data copy and synthesis in Figure 5, the carrier wipeoff is performed. Since shared memory space is not sufficient to store sine and cosine values in addition to data samples and C/A code values, the necessary sine and cosine values are directly accessed from global memory. Sine and cosine values are not numerically calculated in our implementation to reduce computational burden. Instead, lookup tables are stored in global memory. These lookup tables have sine and cosine values within a possible carrier frequency range at baseband (−10 kHz to 10 kHz) with a 10 Hz resolution. The reason to use the limited shared memory to store C/A code values rather than sine and cosine values is that each C/A code value is accessed three times–once for an early, a prompt, and a late replica while sine and cosine values need to be accessed once. Thus, it is more beneficial in terms of reducing total global memory access to use shared memory for C/A code values rather than sine and cosine values.

Once the carrier wipeoff is completed, the code wipeoff is performed (Figure 5). At this stage, the C/A code values and the inphase and quadrature samples after the carrier wipeoff are already stored in shared memory. Thus, their access latency is roughly 100 times lower than global memory access if there are no shared memory bank conflicts (Section 3.2.2 of [43]). The GTX 480 GPU has 32 shared memory banks (Table 3). Since consecutive 32-bit words are assigned to consecutive banks, consecutive data samples (32-bit floating-point numbers in our SDR) are assigned to consecutive banks. If different threads access different banks simultaneously, bank conflicts do not occur and shared memory accesses can be parallelized. By appropriate thread indexing for linear addressing (consecutive threads access consecutive 32-bit data samples as in Figure F-2 of [36]), we remove shared memory bank conflicts. Now the code wipeoff operation can be done very efficiently without further global memory access or bank-conflicted shared memory access.

#### 3.2.4. Parallel Reduction in Reallocated Shared Memory

**Figure 7.**
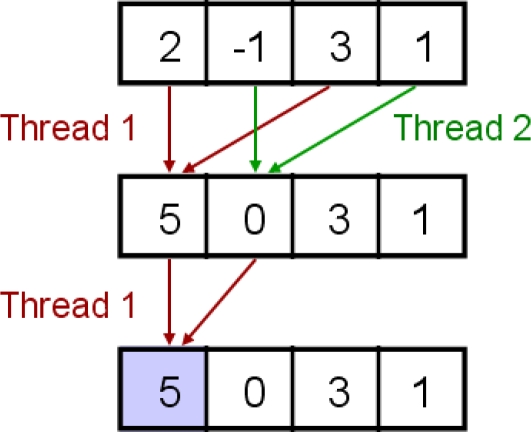
Simple example of parallel reduction for the summation of four numbers.

Once the carrier and code wipeoff is completed, the input inphase and quadrature data and a C/A code replica in shared memory are no longer used by the parallel correlator. Hence, the valuable resource, shared memory, is reallocated for the next operation. The next operation to complete correlation in Figure 5 is to sum the obtained values after the carrier and code wipeoff. Parallel reduction is a well-known parallel algorithm for fast summation of many values. Figure 7 shows a simple example of parallel reduction for summation of four numbers. Thread 1 adds numbers 2 and 3, and thread 2 adds numbers −1 and 1 at the same instruction cycle in this example. Note that adjacent threads accesses adjacent numbers (*i.e.*, 2 and −1, or 3 and 1) to prevent shared memory bank conflicts. If thread 1 adds numbers 2 and −1, and thread 2 adds numbers 3 and 1, bank conflicts would occur. (See [45] for detailed discussion of optimized parallel reduction algorithms in GPU.)

Before the parallel reduction in Figure 5, each thread sums three values and assigns the sum to the reallocated shared memory. Thus, 256 numbers per thread block are ready for parallel reduction. The number of samples for parallel reduction should be a power of 2 because a half of the numbers are added to the other half of the numbers every instruction cycle. At the first instruction cycle, threads 1 through 128 add two numbers each. At the second instruction cycle, threads 1 through 64 add two numbers each, and so forth. Hence, 256 numbers are added after eight instruction cycles in this parallel algorithm, which is much faster than 255 instruction cycles of serial summation. This parallel reduction should be performed in shared memory rather than global memory because the algorithm requires repeated memory access. Once the summation is completed, the accumulated number is stored as the first element of the 256-element array used for parallel reduction.

Since 27 thread blocks perform the correlation operation for one tracking channel, the result of parallel reduction of each thread block should be added together for a final result. Different thread blocks cannot communicate each other via shared memory. Thus, those numbers are atomically added to a variable in global memory in our implementation. Atomic addition of 32-bit floating-point numbers in global memory is supported by compute capability 2.x (Table 3). Atomic addition is a serial operation, but serial addition of 27 numbers from 27 thread blocks is not computationally intensive. Computationally intensive summations are already completed by parallel reduction in each thread block. The result of the parallel correlation in Figure 5 is six numbers (an early, a prompt, and a late correlation for inphase and quadrature samples) per tracking channel. The memory copy latency of these six numbers from global memory to host memory is negligible. The GPU kernel for the carrier and code wipeoff is executed for each 1 ms data block. Its execution time for 1 ms data is about 0.59 ms (averaged over 10,000 measurements).

### 3.3. Hardware Parallelism

**Figure 8.**
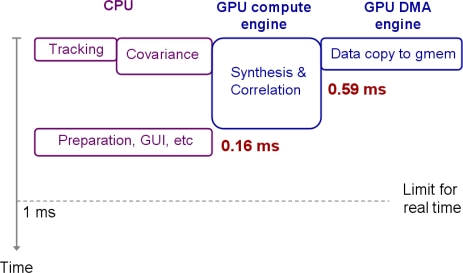
Hardware parallelism with CPU, GPU compute engine, and GPU DMA engine in addition to thread-level parallelism by multi-core CPU and GPU. Total processing time for 1 ms data is about 0.75 ms.

The GPU kernel performing the data synthesis and the carrier and code wipeoff handles (1,080*N* + 360*N*)/1,504*N* ≈ 96% computational load of the CRPA processing. The remaining 64*N*/1504*N* ≈ 4% computation is for the signal covariance calculation (Table 2). Since the CPU and GPU are different hardware, they can perform different tasks in parallel. The covariance calculation can be also performed by another GPU kernel, but the quad-core CPU (Intel Core i7 950) is powerful enough to complete the covariance calculation while the GPU kernel performs the data synthesis and the carrier and code wipeoff. Thus, we assign the covariance calculation to CPU and run it with GPU in parallel (Figure 8). For parallel processing on the quad-core CPU, we generate 10 CPU threads for the covariance calculation. The covariance matrix X^*^X*^T^* in Equation (4) is a Hermitian matrix (*i.e.*, it is equal to its own conjugate transpose). For a four-element antenna array, X^*^X*^T^* is a 4-by-4 matrix. Since it is a Hermitian matrix, we need to calculate the upper triangular part only (*i.e.*, 10 numbers) and take complex conjugate of calculated numbers to obtain the lower triangular part. Thus, we assign one CPU thread for calculating each of 10 numbers in the upper triangular part of the covariance matrix.

Further, we assign five CPU threads for signal tracking operations based on the correlation results from a previous millisecond (1 thread per antenna and 1 thread for beamsteering channels result in 5 threads). Although we generate 15 threads for the covariance calculation and signal tracking, the quad-core CPU can run up to eight threads simultaneously by its hyper-threading technology. Hence, the generated 15 threads run together only by splitting CPU time. The multi-threaded covariance calculation on CPU is further optimized by single instruction multiple data (SIMD) instructions. The execution time of 10 CPU threads for the covariance calculation is about 0.29 ms. The tracking operation is relatively light weight, and the execution time of five CPU threads for tracking is about 0.07 ms. Figure 8 shows the timing diagram of the parallel operations by CPU and GPU. Although the tracking and the covariance calculation by CPU seem to run independently and simultaneously in this figure, it is not exactly true. As already explained, five threads for tracking and 10 threads for covariance calculation share CPU time. However, the CPU operations and the GPU operations run completely in parallel because they are different hardware.

Another important hardware parallelism exists within the graphics card. The input data should be copied from host memory to device global memory first before any GPU operations, and the latency of this memory copy is significant due to limited PCI-E bandwidth (Figure 6). However, NVIDIA GPUs have dedicated DMA engines. Thus, the latency of the host-to-device memory copy by the GPU DMA engine can be completely hidden behind the GPU computation provided that the memory copy requires less execution time than the computation. In order to realize this parallelism, we implement two data buffers (so called “ping-pong” buffers) and perform the GPU computation on 1 ms data within one buffer while the DMA engine copies next 1 ms data to the other buffer and vice versa. In addition, two CUDA streams should be created (one for data copy and the other for computation) and GPU memories should be allocated as page-locked memory to utilize asynchronous CUDA functions (see [46] for a detailed discussion of overlapping data copy and computation).

Page-locked memory allows faster memory access as an additional benefit. Since the GPU DMA engine can directly access page-locked memory, the latency of page-locked memory is lower than the latency of conventional pageable memory. CUDA 4.0 Toolkit released in March 2011 [47] allows a simple way to utilize page-locked memory by a new function called ‘cudaHostRegister’. Once existing variables allocated in host memory are registered using this function, they can be accessed faster as page-locked memory. Special memory allocations using ‘cudaHostAlloc’ as in the previous CUDA versions, for example, are not necessary. Thus, the benefit of page-locked memory can be appreciated without significant modification of existing codes.

After the parallel executions of CPU, GPU compute engine, and GPU DMA engine on 1 ms incoming data, CPU needs to prepare next 1 ms data by memory copy within ring buffer in host memory, etc. In addition, CPU needs to update graphical user interface (GUI) of the SDR. These remaining tasks take about 0.16 ms as shown in Figure 8. The total processing time for 1 ms data is about 0.75 ms. Hence, the GPS SDR for CRPA demonstrates real-time capability with sufficient timing margin.

### 3.4. Optimization Considerations

This subsection further discusses the performance of our GPU kernel and possible optimization strategies for a generic GPU kernel for future GPS SDR developments. As mentioned in Section 3.2, GPU has weakness on tasks requiring high global memory access. It is a usual design assumption that the performance of a GPU kernel would be limited by memory access (memory-bound) rather than computation intensity (compute-bound). Thus, it is a good design practice to start from optimizing global memory access by aligned and coalesced memory access and an efficient use of shared memory. Generating large number of threads and providing high GPU occupancy are also recommended to hide memory and register access latency. Once global memory access is optimized, necessary computations would be implemented on top of the memory access codes. Execution paths of 32 threads within the same warp should be identical for maximizing computational efficiency.

In order to check the limiting factor of the performance of our GPU kernel, all computation codes in the kernel are commented out and the execution time of memory access codes alone is measured. The memory access alone takes about 0.42 ms for 1 ms input data (remember that the original kernel with memory access and computation takes about 0.59 ms). After that, the global memory access codes are commented out and the execution time of computation codes alone is measured. The computation alone without global memory access takes about 0.50 ms. Therefore, our kernel is compute-bound rather than memory-bound, which means further optimization of memory access may not reduce the kernel execution time. Note that the global memory access latency is well-hidden in our GPU kernel. Adding the global memory access codes to the computation codes increases kernel execution time by only 0.09 ms, even though the global memory access alone takes about 0.42 ms. GPUs hide memory latency by fast thread context switching and many more threads than available CUDA cores. In other words, CUDA cores do not wait for memory reads of certain threads but perform instructions on other available threads until the memory read is completed.

If a generic GPU kernel for a GPS SDR is memory-bound, several optimization schemes could be considered. Global memory access can be reduced with the cost of increased computation. For example, we use sine and cosine tables in global memory to reduce computational burden, but if a kernel is memory-bound, this approach is not recommended. Instead, sine and cosine values should be calculated by threads in order to reduce global memory access. A similar approach is possible for the C/A code tables. We copy necessary C/A code values from lookup tables in global memory to shared memory before the code wipeoff. Instead, the C/A code table can be stored in constant memory as in [32]. Constant memory is cacheable read-only memory. Although constant memory access is faster than global memory access, it is a very limited resource (64 KB per device). In order to fit the C/A code table into this small memory space, Knezevic *et al.* [32] compressed the table. Thus, the C/A code values need to be decompressed before the code wipeoff operation, which requires extra-computations. This is a good optimization strategy for a memory-bound GPU correlation kernel, but our kernel is compute-bound and it is undesirable to increase computational intensity for reducing global memory access.

There are also several optimization strategies for a compute-bound kernel for GPS SDR. All GPU computations in our implementation are 32-bit floating-point operations. One may think that replacing floating-point operations to integer operations could provide computational benefit. In fact, GPUs of compute capability 2.0 can perform 32 32-bit floating-point operations (add, multiply, and multiply-and-add) per clock cycle per multiprocessor and the same number of 32-bit integer operations (add, logical operation) per clock cycle per multiprocessor (Table 5.1 of [36]). For 32-bit integer multiply or multiply-and-add, the GPUs can perform only 16 operations. Thus, replacing 32-bit floating-point operations to 32-bit integer operations would not provide any computational benefit. CUDA-enabled GPUs do not natively support 16-bit integer operations, so 16-bit integer operations do not provide benefit either. GPUs of compute capability 1.x natively support 24-bit integer multiply ‘–mul24’, but GPUs of compute capability 2.x natively support 32-bit integer multiply instead. Hence, applying 24-bit integer multiply instead of 32-bit multiply in devices of compute capability 2.x actually decreases performance.

As a more promising optimization strategy for a compute-bound kernel, Lee *et al.* [48] suggested to use shared memory to reduce register usage and consequently increase GPU occupancy. Resister usage is the limiting factor of the occupancy of our kernel as discussed in Section 3.2. In order to reduce one 32-bit register use per thread, we need 32 bit × 256 thread = 1,024 Byte shared memory per thread block. However, the remaining shared memory space per thread block in our kernel is only 9.6 KB − 9.3 KB = 0.3 KB. Thus, this strategy would not be effective in our case.

Another suggestion in [48] for a compute-bound kernel is to assign one big thread block to each multiprocessor so that the thread block can use the whole resource of the multiprocessor. If we can assign one thread block with 1,280 threads per multiprocessor instead of five thread blocks with 256 threads each, available shared memory space for the one big thread block would be 0.3 KB/block × 5 block = 1.5 KB. This space can store about 125 inphase samples, quadrature samples, and C/A code values. However, the maximum number of threads per thread block is 1,024 (Table 3), so it is not possible to assign 1,280 threads to one block. We may assign two thread blocks with 640 threads each. Then, available shared memory space for each block would be 1.5 KB / 2 block=0.75 KB / block. This space can hold about 62 inphase samples, quadrature samples, and C/A code values. Although we can possibly process about 62 more samples per thread block with this configuration, 62 is not a multiple of the number of threads per thread block (*i.e.*, 640 in this case). Thus, it would be difficult to prepare data for parallel reduction without divergent warps. If some warps are divergent, performance could decrease after changing the design to use two thread blocks.

Although the aforementioned optimization strategies may not be beneficial to our GPU kernel, we can further improve the performance of our GPS SDR by utilizing the CPU more efficiently. The preparation and GUI operations in Figure 8 do not need to wait until the GPU kernel finishes its operations. If the preparation and GUI operations start right after the covariance calculation, the whole CPU operations would take about 0.29 ms (covariance) + 0.16 ms (preparation, GUI, *etc.*) = 0.45 ms. Therefore, the CPU operations (0.45 ms) can be completely hidden behind the GPU operations (0.59 ms). We have not yet implemented this idea because the current GPS SDR for CRPA fully serves our purpose and the code modifications to incorporate this idea are not trivial in our current code base.

Performance improvement by use of newer hardware is always expected. The GTX 480 GPU released in March 2010 that we use for this work has 480 CUDA cores and 1,401 MHz processor clock [44]. The GTX 580 GPU released in November 2010 has 512 CUDA cores and 1,544 MHz processor clock [49]. As in Equation (7), the theoretical maximum throughput of the GTX 580 is calculated in Equation (8), which shows about 18% improvement over the throughput of GTX 480:

(8) 512 cores×1544 MHz×2 flop/core/cycle=1.58 Tflops

Thus, if we run our compute-bound kernel in GTX 580, its execution time should be decreased (if the kernel is memory-bound, more operations per clock cycle do not necessarily improve its performance). This new hardware, GTX 580, also provides benefit for a memory-bound kernel by its faster memory clock (2,004 MHz). Its theoretical maximum global memory bandwidth is calculated in Equation (9). Comparing with Equation (6), about 8% improvement over the bandwidth of GTX 480 is achieved:

(9) $$384\;\text{bit}\times \frac{1}{8}\text{Byte}/\text{bit}\times 2004\;\text{MHz}\times 2=192.4\;\text{GB}/s$$

We demonstrate a real-time capability of our GPS SDR for CRPA using single CPU and single GPU. Nevertheless, if GPU throughput is really a concern for future GPU-based GPS SDR developments for other applications, multiple graphics cards can share the burden. The CUDA architecture is scalable and the CUDA 4.0 Toolkit released in March 2011 provides significantly improved multi-GPU programming features [47].

## 4. Anti-Jam Capability of GPS SDR for CRPA

**Figure 9.**
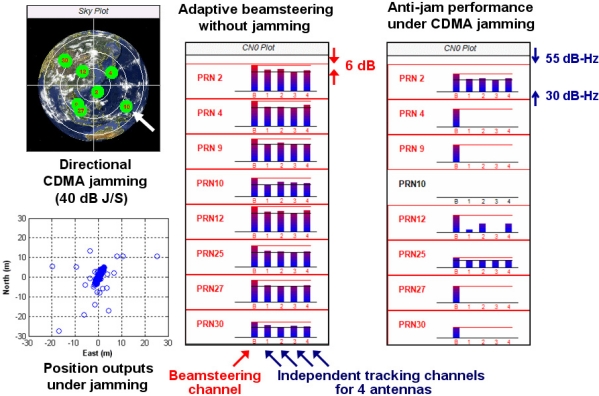
Anti-jam capability of GPS SDR for CRPA under synthesized CDMA jamming. The SDR is capable of tracking 12 satellites but eight satellites are shown here because eight satellites are visible at this epoch.

This section demonstrates the anti-jam capability of our GPS SDR for CRPA under a synthetic wideband jammer. Live GPS L1 signals through a four-element antenna array are IF-sampled by four USRP2s and recorded in four SSDs. As mentioned in Section 3.1, we record GPS L1 data with 40 Msps rate to demonstrate the SDR’s computational capability for future GPS L5 signal processing. Synthesized code division multiple access (CDMA) jamming signals are added to the recorded L1 signals by post-processing. Wideband CDMA jammers are more challenging to GPS receivers than narrowband continuous-wave, or CW, jammers. The jamming direction is selected as the direction of GPS signals from PRN 10 (Figure 9). This CDMA jammer is simulated using PRN 168, which is currently unused by any satellite, and the interference signal is amplified to have 40 dB jamming-to-signal-power ratio (J/S).

Figure 9 shows parts of the captured output window of the SDR. Without adding the synthesized CDMA jammer, all tracking channels of the SDR track valid satellites in view as the C/N_0_ plot of Figure 9 (middle) demonstrates. There are five tracking channels for each satellite. The first channel of each satellite in Figure 9 is the tracking channel for composite signal beamsteered to that satellite. The remaining four channels are the individual tracking channels for each of the four antennas for that satellite. The beamsteering channels of all satellites show about 6 dB C/N_0_ enhancements due to the increased gain resulting from utilizing signals from four antennas rather than one antenna. These directional beams toward satellites provide benefits, such as mitigation of multipath errors, even under nominal conditions without GPS jamming.

Once the simulated CDMA jammer is injected, most single antenna channels lose tracking as in Figure 9 (right). (The SDR does not show C/N_0_ of lost channels.) We modulate alternating 1 s and −1 s in the navigation data of the jamming signals in order to check if the SDR tracks valid satellites instead of the jammer. In fact, the single antenna channels of PRN 25 track the jammer instead of the correct satellite partly due to high cross correlation. Nevertheless, the beamsteering channels of all satellites including PRN 25 still track the correct satellites, and the receiver outputs correct position solutions. (PRN 10 is an exception because the jammer is injected in this direction.)

Although our GPS SDR for CRPA rejects moderate to high levels of interference in the tests with live signals and synthetic interference, the saturation effects of the analog front-end due to real jammers cannot be studied in this way. Analytical and numerical studies neglecting these effects can be somewhat optimistic for the real-world performance of the GPS SDR for CRPA. To evaluate the saturation effects, live over-the-air GPS jamming tests, which are difficult to perform due to regulations, are necessary in the future.

## 5. Conclusions

In order to increase the jamming resistance of GPS sensors, we have designed a GPS SDR for a four-element CRPA array. This GPS SDR is capable of real-time beamsteering toward 12 GPS satellites with a sampling rate of 40 Msps for future GPS L5 signals. The on-the-fly phase calibration of this paper provides a simple way to align phases between multiple antenna elements of a stationary GPS CRPA. The computational requirement of our CRPA processing has not been satisfied by previous GPS SDRs in the literature. We propose an optimal GPS SDR architecture using CPU and GPU and maximize the usage of their parallel computation capabilities including thread-level parallelism and hardware parallelism. General-purpose GPU technology is relatively new and actively evolving. Our GPU kernel is specifically optimized for the recent development of a new GPU architecture with compute capability 2.x and a new CUDA 4.0 Toolkit. Since design and optimization strategies using this new generation GPU are not widely available in the GPS sensors community, this paper tries to provide sufficient details for future GPS SDR developments for other applications.

Although this paper focuses on the design of a GPU kernel, a modern multi-core CPU is powerful enough to share a large portion of the computational load. Modern processors are evolving towards increasing the number of cores rather than the speed of processing clocks. As a result, modern processors demonstrate very high instruction throughputs, but users need to parallelize their computations in order to properly utilize the parallel resources. Multi-threading on CPU and SIMD operations by each thread, as implemented in our SDR, are essential to fully appreciate the CPU resources.

The GPS CRPA technology is more mature in the military market, but the technology is mostly confidential and unreleased for civil applications. However, GPS RFI is not just a military concern and some civil applications, such as GPS-based aviation, require certain level of resistance to GPS RFI. This paper demonstrates that GPS CRPA technology can be implemented using cost-efficient COTS hardware and processors, which envisions wide applications of this technology.

## Figures and Tables

**Table 1.** Comparison of GPUs which have been used for GPS SDRs in the literature. The specifications of GPUs are selected from Table A-1 of [36].

	**Compute Capability**	**Number of Multiprocessors**	**Number of CUDA Cores**	**Previous GPU-based GPS SDR Development**
GeForce 8800 GTX	1.0	16	128	8 channels, 40 Msps, 8-bit resolution [32]
GeForce GTX 280	1.3	30	240	12 channels, 8 Msps, 4-bit resolution [33]
GeForce GTX 285	1.3	30	240	150 channels, 5 Msps, 14-bit resolution [34]
GeForce GTX 480	2.0	15	480	Acquisition only [35]

**Table 2.** Comparison of the required number of multiplications and additions for the GPS SDR for CRPA in this paper and a conventional GPS L1(C/A) SDR. N is the number of inphase or quadrature samples per millisecond (N_L5_ = 10 N_L1_). N_L5_ = 20,000 is used in this work.

	
	**GPS SDR for four-element CRPA**	**Conventional GPS SDR**
Carrier and code wipeoff	1,080 N_L5_	216 N_L1_
Data synthesis by weighting	360 N_L5_	N/A
Covariance calculation	64 N_L5_	N/A

**Table 3.** Technical specifications and feature support per compute capability (Selected from Tables F-1 and F-2 of [36]).

	Compute Capability				
	1.0	1.1	1.2	1.3	2.x
Maximum number of resident thread blocks per multiprocessor	8				
Maximum number of resident warps per multiprocessor	24		32		48
Maximum number of resident threads per multiprocessor	768		1024		1536
Number of 32-bit registers per multiprocessor	8 K		16 K		32 K
Maximum amount of shared memory per multiprocessor	16 KB				48 KB
Number of shared memory banks	16				32
Amount of local memory per thread	16 KB				512 KB
Maximum number of threads per block	512				1024
Atomic addition operating on 32-bit floating point values in global and shared memory	No				Yes
